# Controlled human drug administration studies are necessary to define the THC-sparing effects of CBD and other cannabis constituents

**DOI:** 10.1038/s41386-023-01538-y

**Published:** 2023-02-01

**Authors:** Elisa Pabon, Ziva D. Cooper

**Affiliations:** 1 UCLA Center for Cannabis and Cannabinoids, Jane and Terry Semel Institute for Neuroscience and Human Behavior, Department of Psychiatry and Biobehavioral Sciences, David Geffen School of Medicine, University of California, Los Angeles, CA USA; 2 Charles R. Drew University of Medicine and Science, Los Angeles, CA USA; 3 Department of Anesthesiology and Perioperative Medicine, David Geffen School of Medicine University of California, Los Angeles, CA USA

**Keywords:** Drug development, Addiction, Human behaviour

Cannabis is the most commonly used drug in the United States after alcohol and nicotine. With ongoing policy changes, cannabis accessibility and use will increase. Cannabis contains phytocannabinoids, terpenes, and flavonoids; concentrations of each vary according to chemovar and cannabis-derived product preparation. The two most studied phytocannabinoids are delta-9-tetrahydrocannabinol (THC) and cannabidiol (CBD). THC is the primary psychoactive constituent contributing to the potential therapeutic and adverse effects of cannabis. Adverse effects attributed to THC include cognitive impairment, intoxication, anxiogenic effects, psychotic-like experiences, abuse liability, and development of cannabis use disorder. These effects increase risks associated with non-medical cannabis use and limit the clinical utility of THC when used for medical purposes. CBD lacks the adverse effects of THC. Early clinical studies point to CBD’s anxiolytic and antipsychotic properties–effects that oppose two risks associated with THC [1]. Hypotheses rooted in preclinical findings have spurred interest in the potential for cannabis constituents to interact with THC, mitigating THC’s risks and enhancing its potential medicinal properties (i.e., THC-sparing effects). To date, the overwhelming share of clinical research probing THC-sparing effects of cannabis constituents focuses on interactions between THC and CBD, which has generated mixed findings. Nonetheless, a significant segment of the cannabis market consists of CBD-THC combination products. These products are commonly sold according to the ratio of CBD to THC doses in each unit; popular ratios range from 1:1 to 50:1 with the suggestion that higher CBD:THC ratios may be “safer.” Here we comment on a recent clinical study probing CBD’s THC-sparing effects, describe how findings impact the field, and highlight future research designed to inform medical and non-medical cannabis use.

In this issue of Neuropsychopharmacology, Englund et al. report on one of the first controlled drug administration studies to systematically assess the THC-sparing effects of co-administered CBD across a range of CBD:THC ratios typically available in commercial markets (0:1, 1:1, 2:1, 3:1) [2]. While holding the THC dose (10 mg) constant and increasing the CBD dose (0, 10, 20, 30 mg), the adverse effects of THC administered alone were defined and the impact of CBD on these endpoints was characterized. The study builds on the authors’ earlier report that pre-treatment of oral CBD (600 mg) attenuates memory impairment and psychotic-like symptoms following intravenous THC administration. The current study extends those findings, examining an ecologically relevant mode of THC administration (inhalation versus intravenous) and probing CBD’s impact on THC effects using commercially available CBD:THC ratios. CBD did not impact THC-induced cognitive impairment, psychotic-like experiences, or ratings of intoxication, anxiety, or drug liking at any dose. These findings do not support the hypothesis that CBD has THC-sparing effects when co-administered according to commonly used doses and routes of administration.

Given preclinical and clinical studies pointing to CBD’s potential THC-sparing effects, the findings were unexpected. However, this investigation adds to the growing literature of controlled drug administration studies that fail to find THC-sparing effects on similar endpoints highlighted here, including intoxication, cognitive effects, and abuse liability [1]. Factors that may contribute to differences observed across studies include varied routes of THC and CBD administration and administration of CBD doses that far exceed those assessed here. For example, a much higher dose of inhaled CBD (400 mg) than used in the current study significantly reduced intoxication associated with THC (8 mg) [3]. Another example of a study design variable that may impact outcomes is frequency of cannabis use among participants. A recent study with volunteers who used cannabis more frequently than those in the current study compared inhaled cannabis with THC only (13.75 mg THC) to cannabis with near-equivalent CBD and THC doses (13.75 mg CBD/13.75 mg THC); cannabis with both CBD and THC elicited lower ratings of anxiety compared to the THC-only cannabis [4]. Given inconsistent findings across studies, research should continue investigating the potential THC-sparing effects of CBD controlling for frequency of cannabis use. Of prime importance is understanding the effects of ecologically relevant modes of administration and CBD-THC dose combinations and ratios. These studies are critical not only to understand the potential THC-sparing effects of CBD but also to identify dose conditions that may increase THC’s adverse effects, as has been observed with low CBD doses (4 mg) in combintion with THC (8 mg) [3].

Much of the controlled drug administration literature related to CBD’s THC-sparing effects focuses on reducing risks associated with THC in healthy participants under acute dosing conditions. However, both non-medical and medical cannabis use likely involve repeated, frequent use. While some observational and preclinical studies suggest that CBD may reduce neuroadaptations associated with repeated THC exposure [5], controlled drug administration studies systematically investigating the effects of repeated CBD and THC co-administration compared to THC alone are lacking. In addition, few studies examine CBD’s potential THC-sparing effects in patient populations, to address whether CBD co-administration reduces THC’s adverse effects, while also improving therapeutic outcomes beyond what is observed when THC is administered alone. Because of widespread availability and medical use of CBD-THC combination products, data from rigorously controlled studies investigating CBD’s potential THC-sparing effects are urgently needed.

Currently, there is little evidence supporting the popular hypothesis that ecologically relevant cannabis products containing higher CBD:THC ratios are “safer.” To better inform medical practice and guide cannabis-related public health and policy decisions, continued investigation into the acute and long-term health effects of CBD-THC combinations is needed. As the field strives to identify and define CBD’s impact on THC-associated health outcomes, other cannabis constituents with potential THC-sparing properties are garnering attention. The pharmacology of specific phytocannabinoids such as cannabinol, cannabigerol, and delta-9-tetrahydrocannabivarin, and terpenes, like myrcene, humulene, and beta-caryophyllene, suggest unique opportunities to explore additional cannabis-based strategies to reduce risks associated with THC and enhance potential therapeutic outcomes. Cannabis-based products with these constituents are already available in many regions of the United States. Controlled drug administration studies similar to that of Englund et al. [2] using ecologically relevant doses and modes of administration will be pivotal in determining the safety of these constituents and characterizing their interactions with THC.
